# Bimodal Biometric Verification Using the Fusion of Palmprint and Infrared Palm-Dorsum Vein Images

**DOI:** 10.3390/s151229856

**Published:** 2015-12-12

**Authors:** Chih-Lung Lin, Shih-Hung Wang, Hsu-Yung Cheng, Kuo-Chin Fan, Wei-Lieh Hsu, Chin-Rong Lai

**Affiliations:** 1 Department of Electronic Engineering, Hwa Hsia University of Technology, 111 Gon Jhuan Rd., Chung Ho dist., New Taipei City 23568, Taiwan; 2 Institute of Computer Science and Information Engineering, National Central University, Taoyuan City 32001, Taiwan; andy52400@gmail.com (S.-H.W.); chengsy@csie.ncu.edu.tw (H.-Y.C.); kcfan.ncu@gmail.com (K.-C.F.); kenlay98@gmail.com (C.-R.L.); 3 Department of Computer Information and Network Engineering, Lunghwa University of Science and Technology, Taoyuan County 33306, Taiwan; Leih@ms2.hinet.net

**Keywords:** biometric verification, palmprint, vein pattern, discrete wavelet transform, image fusion, support vector machine

## Abstract

In this paper, we present a reliable and robust biometric verification method based on bimodal physiological characteristics of palms, including the palmprint and palm-dorsum vein patterns. The proposed method consists of five steps: (1) automatically aligning and cropping the same region of interest from different palm or palm-dorsum images; (2) applying the digital wavelet transform and inverse wavelet transform to fuse palmprint and vein pattern images; (3) extracting the line-like features (LLFs) from the fused image; (4) obtaining multiresolution representations of the LLFs by using a multiresolution filter; and (5) using a support vector machine to verify the multiresolution representations of the LLFs. The proposed method possesses four advantages: first, both modal images are captured in peg-free scenarios to improve the user-friendliness of the verification device. Second, palmprint and vein pattern images are captured using a low-resolution digital scanner and infrared (IR) camera. The use of low-resolution images results in a smaller database. In addition, the vein pattern images are captured through the invisible IR spectrum, which improves antispoofing. Third, since the physiological characteristics of palmprint and vein pattern images are different, a hybrid fusing rule can be introduced to fuse the decomposition coefficients of different bands. The proposed method fuses decomposition coefficients at different decomposed levels, with different image sizes, captured from different sensor devices. Finally, the proposed method operates automatically and hence no parameters need to be set manually. Three thousand palmprint images and 3000 vein pattern images were collected from 100 volunteers to verify the validity of the proposed method. The results show a false rejection rate of 1.20% and a false acceptance rate of 1.56%. It demonstrates the validity and excellent performance of our proposed method comparing to other methods.

## 1. Introduction

Unimodal biometric verification based on physiological characteristics such as the iris, retina, face, fingerprint, palmprint, hand geometry, and vein patterns are increasingly demanded for security systems. However, they have still many challenges, such as limited information, limited feature representations and weak antispoofing capabilities. Thus, achieving a high accuracy rate in unimodal biometric verification remains a challenge. As a result, bimodal biometric verification was developed. The greatest advantage of bimodal biometric verification is that multiple information points can be acquired from different modal characteristics.

This paper proposes a biometric verification method based on two physiological characteristics: the palmprint and palm veins. A palmprint image is rich in line features such as wrinkles, ridges, and principal lines. A palm vein image contains rich texture features shown in its vein patterns. The proposed method fuses these two modal images and hence results in richer and complementary information of one or more biometric characteristics. Many techniques can be used to perform the fusion, including the well-known discrete wavelet transform (DWT) and inverse discrete wavelet transform (IDWT). The proposed method uses DWT and IDWT to fuse palmprint and palm vein images.

Biometric verification methods using palmprint features have been developed over the past decades. Jain *et al.* [1] identified 14 different biometric features that could be used to verify hand shapes by using deformable matching technology. Huang *et al.* [2] proposed a palmprint verification technique based on principal lines. These principal lines were extracted using a modified finite Radon transform, and a binary edge map was used for representation. Han *et al.* [3] extracted features such as finger length, finger width, and palmprints to be used as inputs for principal component analysis. Zhang *et al.* [4] applied the line-matching idea to print matching. They transferred palmprints to line sections and used these features to identify people. Lu *et al.* [5] applied a Karhunen–Loeve transformation to transform an original palmprint into an eigenpalm, which could represent the principal components of the palmprint. Then, the weighted Euclidean distance classifier was applied for palmprint recognition. In another study, texture-based codes such as the competitive code [6] and the orthogonal line ordinal feature [7] were used to extract the orientation of lines which exhibit state-of-the-art performance in palmprint recognition. Kong *et al.* [8] applied a two-dimensional Gabor filter to obtain texture information from palmprint images. Two palmprint images were compared in terms of their Hamming distance of texture information. Zhang *et al.* [9] obtained a palmprint feature by using a locality-preserving projection based on a wavelet transform. Lin *et al.* [10] presented a palmprint verification method that involved using a bifeature, palmprint feature-point number and a histogram of oriented gradient. Lu *et al.* [11] proposed a system of capturing palm images in peg-free scenarios by using a low-cost and low-resolution digital scanner. Lin *et al.* [12] applied a hierarchical decomposition mechanism to extract principal palmprint features inside the region of interest (ROI), which included directional and multiresolution decompositions. They used a normalized correlation function to evaluate similarities. Han *et al.* [13] used four Sobel operators and complex morphological operators to extract the features of a palmprint, and applied the backpropagation neural network and template matching with a normalized correlation function to verify persons.

Compared with the palmprint, the use of palm veins is a relatively new hand-based biometric trend. MacGregor *et al.* [14] were the first to present a system for personal identification using palm veins. Im *et al.* [15] employed a charge coupled device (CCD) camera to capture vein pattern images. Their research focused on implementing fixed-point operations to improve verification speeds and reduce hardware costs. Mirmohamadsadeghi *et al.* [16] investigated two new feature extraction approaches based on a variety of multiscale, local binary patterns and high-order local derivative patterns to identify the optimal descriptors for palm veins. Lin *et al.* [17] obtained multiresolution representations of images with feature points of the vein patterns (FPVPs) by using multiple multiresolution filters that extracted the dominant points by filtering the miscellaneous features for each FPVP. Shahin *et al.* [18] proposed biometric authentication using a fast spatial correlation of hand vein patterns, and designed a system with a near infrared cold source to provide back-of-hand illumination. Wang *et al.* [19] combined support vector machines (SVMs) with a k-nearest neighbors algorithm and a minimum distance classifier for palmprint and palm-vein feature matching. Recently, the effectiveness of finger vein recognition was proved by Liu *et al.* [20] using a novel point manifold distance metric. 

Bimodal biometrics have been deployed with particular fusion schemes, including sensor-level, feature-level, and match-score level fusions. Wang *et al.* [21] fused palmprint and palm-vein images and proposed a Laplacian palm representation, which attempts to preserve local characteristics. Kisku *et al.* [22] used a few selected wavelet fusion rules to fuse biometric face and palmprint images at the sensor level. The technique proposed in this paper efficiently minimizes irrelevant distinct variability in the different biometric modalities and their characteristics by performing the fusion of biometric images at the sensor level.

**Figure 1:**
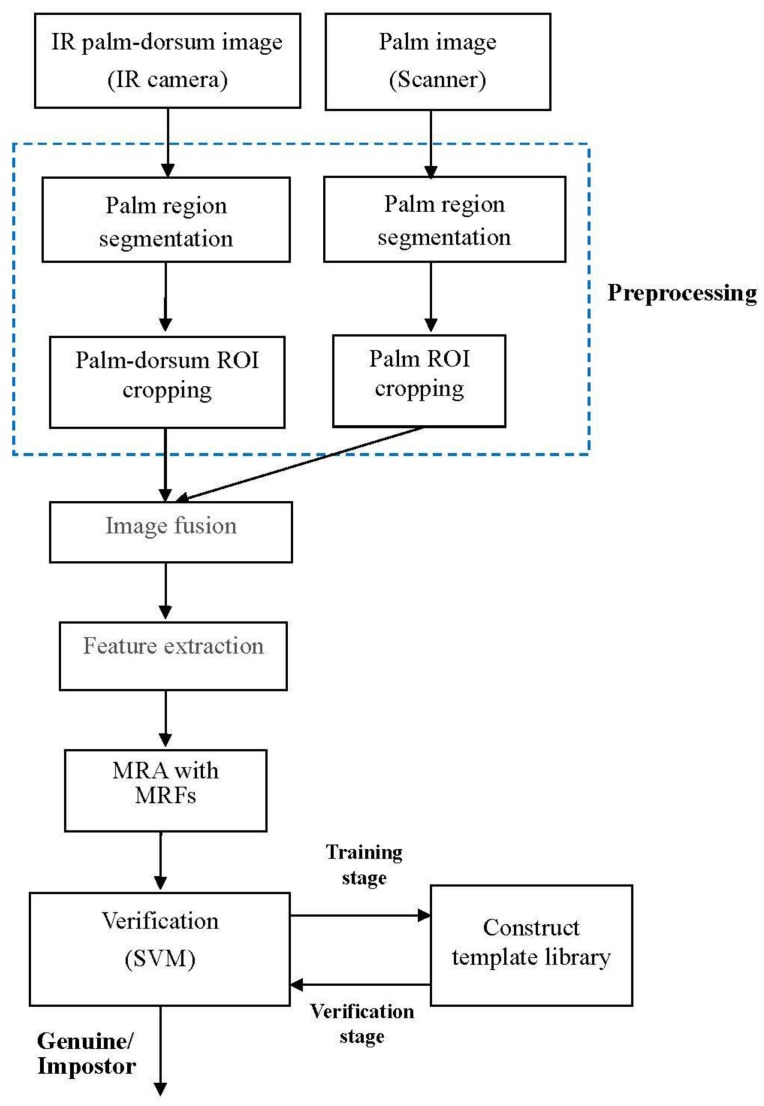
Block diagram of the proposed method.

The proposed method adopts two biometric modals: the palmprint and palm-dorsum vein. A function block diagram is shown in Figure 1. The method is composed of five stages: image acquisition, preprocessing, image fusion, feature extraction, and multiresolution analysis and verification. In the image acquisition stage, a digital scanner and infrared (IR) camera were applied to capture palm and palm-dorsum images. The resolution of the digital scanner used in this study was 100 dpi and that of the IR camera was 320 × 240 pixels. One hundred volunteers were used to capture 3000 palmprint and 3000 vein-pattern images. The preprocessing stage included palm region segmentation, the locating of finger webs, and ROI localization. In the image fusion stage, DWT and IDWT were applied to fuse the two ROI images—the palmprint and vein pattern—into one new fused image. Iterative histogram thresholding was employed to extract line-like features (LLFs) from the fused images. The extracted LLFs were analyzed using a multiresolution filter to obtain the multiresolution representations. Finally, an SVM is adopted to perform the verification between reference templates and testing images.

The rest of this paper is organized as follows: Section 2 describes the preprocessing procedures, which include palm region segmentation, the locating of finger webs, and ROI localization and alignment. Section 3 describes the process of image fusion based on DWT and IDWT. In Section 4, LLF extraction, iterative histogram thresholding, and multiresolution analysis with the multiresolution filter are described. The mechanism of verification based on SVM is demonstrated in Section 5. Section 6 presents the results to verify the validity of the proposed method. Finally, concluding remarks are presented in Section 7.

## 2. Preprocessing

Image preprocessing is necessary to attain high accuracy in biometric verification. The main goal of image preprocessing is to extract the same region from different palm images captured from the same person known as the ROI. Image preprocessing consists of three steps. First, palm region segmentation separates the background from the palm region. Second, the finger-web locations are identified as reference points. Finally, the square ROI is cropped and aligned according to the finger-web points. The details of each step are described in the following subsections.

### 2.1. Palm Region Segmentation

**Figure 2:**
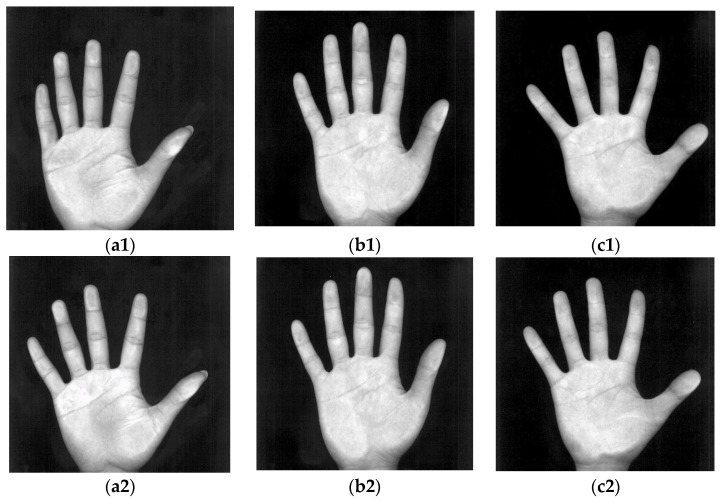

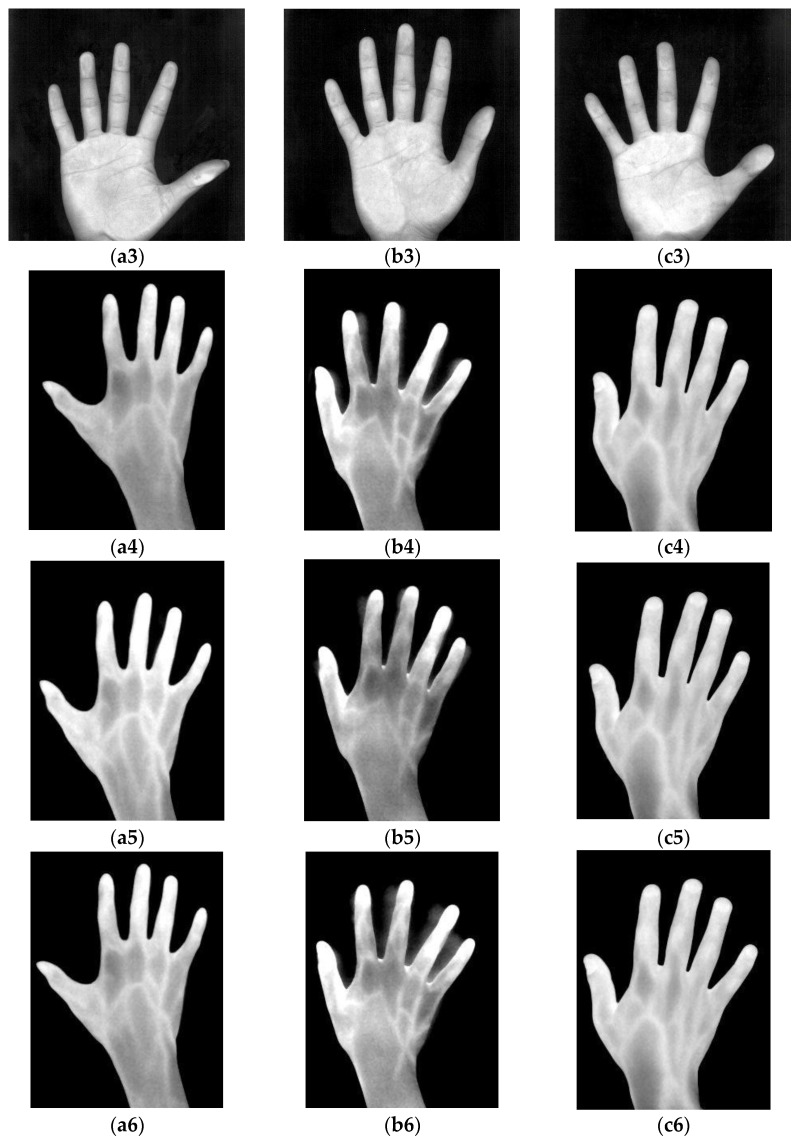
(**a1**)–(**a3**), (**b1**)–(**b3**) and (**c1**)–(**c3**) show the palm images captured by a digital scanner; (**a4**)–(**a6**), (**b4**)–(**b6**) and (**c4**)–(**c6**) show the IR palm-dorsum images captured by an IR camera. Different palm images and IR palm-dorsum images in each column were captured peg-free from one person.

Figure 2 shows palm images captured by the digital scanner at a low resolution and IR palm-dorsum images captured by an IR camera. The palm images have a high quality and low noise. In Figure 2, the images (a1)–(a3), (b1)–(b3), and (c1)–(c3) were each captured peg-free from one person. The proposed method uses an IR camera to capture palm-dorsum images. A crucial property of IR imaging is that the gray levels of pixels change monotonically according to the temperature of the object in the image. Since the temperature of a vein is higher than that of the skin, a vein shows a high gray level in an IR palm-dorsum image which can be distinguished from the low gray level of skin. Figure 2 shows IR palm-dorsum images captured by the IR camera. In Figure 2, the images (a4)–(a6), (b4)–(b6), and (c4)–(c6), were each captured peg-free from one person. As shown in Figure 2, the images contain two parts: the palm region and the black background. For segmenting the palm region, a histogram can provide valuable information. The histograms of these images convey a typical bimodal distribution.

**Figure 3:**
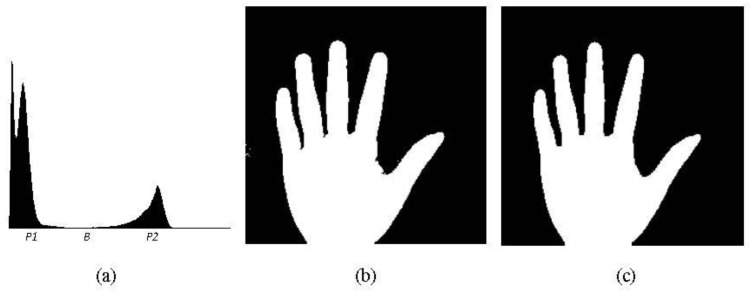
(**a**) Histogram of the palm image from Figure 2a1. The vertical and horizontal axes represent pixel numbers and grey values, respectively. *P1* and *P2* are gray values with the local maximum pixel numbers, whereas *B* is the gray value with the local minimum pixel number. *B* is selected as the threshold to segment the palm region; (**b**) Binarized image of the palm region segmented using threshold *B*, which was selected by the mode method; (**c**) Resulting image of the binary palm image from (**b**) after processing by dilation (twice) and erosion (twice).

Figure 3a shows the histogram of the palm image from Figure 2a1 where the vertical and horizontal axes represent pixel numbers and gray values, respectively. Although the histogram of each palm image shows a typical bimodal distribution, a small difference still exists. To determine a suitable threshold to segment the palm region, an adaptive thresholding process is needed. Many techniques of thresholding are possible, including the mode, P-tile, mean value, and Otsu methods. In our work, the mode method [23] is employed to select the threshold for segmenting the palm region. The mode method selects the gray value with the local minimum pixel number between the two gray values with local maxima. As shown in the histogram of Figure 3a where *P1* and *P2* are the gray values with the local maximum pixel numbers and *B* is the gray value with the local minimum pixel number, *B* will be selected as the threshold to segment the palm region. With the selected threshold, the palm region is segmented from the palm image. The resulting binarized image is shown in Figure 3b. The palm shape is distinctly segmented from the original image, showing that the mode method can efficiently divide palm images into the palm region and the background.

After using the mode method to binarize the palm images, many small broken parts in the boundaries of the palm regions remained, including fingernail and background noises. These broken parts could cause difficulties in later image processing procedures. Therefore, morphological operations are adopted to repair the broken parts.

The well-known morphological theory [24] is frequently used in image processing. A morphological operation explores an image with a template called a structuring element. After applying the morphological operation, a binary image outputs a new binary image. The value of the output image is defined by a comparison with the structuring element and the corresponding neighborhood of pixels. The structuring element is a small binary image, and can be of any shape and size. There are two major morphological operations: dilation and erosion. After dilation, the holes enclosed by a single region and gaps between different regions become smaller, and small intrusions on the boundaries of a region are filled. With erosion, the holes and gaps between different regions become larger, and small details are eliminated. In general, dilation increases the pixels around the boundaries of image objects and erosion reduces the pixels. The equations of dilation and erosion are as follows:

(1) $$\text{A}⊕\text{B}=\cup_{\beta \in \text{B}}\left(\text{A}+\beta \right)$$

(2) $$\text{A}⊖\left(-\text{B}\right)=\cap_{\beta \in \text{B}}\left(\text{A}-\beta \right)$$

where A is the image, B is the structuring element, and β is a pixel belonging to B, −B means “not B”.

The proposed method applies the dilation twice before performing the erosion twice. With these morphological operations, the palm shape of the image becomes more completed. The broken parts are repaired and the noise is removed. The result is shown in Figure 3c.

### 2.2. Locating Finger Webs and ROI Cropping

The palm and IR palm-dorsum images were captured under peg-free scenarios. This increases user-friendliness but results in varied palm locations and rotation angles across images. Biometric features (*i.e.*, the palmprint and vein pattern) extracted from the same region in different images are crucial to increasing verification accuracy and reliability for many biometric recognition systems. The cropped region is known as the ROI. The ROI must be aligned to the same position in different palm or palm-dorsum regions to ensure the stability of the extracted features. The ROI also has a significant influence on the accuracy of verification. However, it is difficult to align the ROI to the same position in different images without using pegs to constrain the palm during image capturing. Varied positions and rotation angles can occur in palm and palm-dorsum images. Some examples of peg-free images captured from one person are shown in Figure 2. These images show that the position and rotation angle of palm and palm-dorsum regions are different for each image.

**Figure 4:**
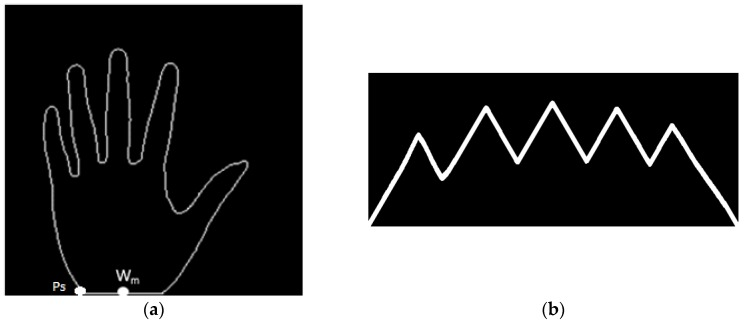
(**a**) Contours of the palm region; (**b**) Distance distribution profile of the palm contours constructed using the Euclidean distances between the wrist middle point *W_M_* and palm contour pixels.

**Figure 5:**
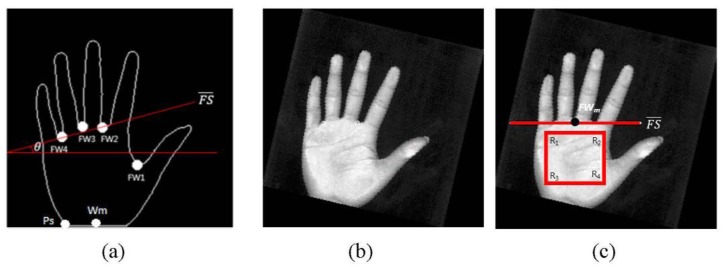
(**a**) Locations of the four finger webs *FW*_1_, *FW*_2_, *FW*_3_, and *FW*_4_. Line $\bar{FS}$ is formed by FW_2_ and FW_4_. The angle *θ* is measured between line $\bar{FS}$ and a horizontal line; (**b**) The resulting image rotated with angle *θ*; (**c**) Location of the ROI.

Our previous study [10,11] was the first to use the second- and fourth-finger webs as datum points to automatically locate and align the ROI. The two finger webs can replace pegs and determine the approximate ROI. The proposed method possesses two significant advantages. First, it can reduce the displacement of the ROI to an acceptable range. Second, the range of palm rotation and translation while acquiring palm images can be eliminated. The finger-web location algorithm here was modified from our previous study [10,11], the procedures of which are stated briefly as follows:

1. The inner-border tracing algorithm [23] is applied to determine the palm contours, and the resulting image is shown in Figure 4a. The process starts from the bottom-left point Ps tracing counterclockwise along the border of the palm shape until rejoining point P_S_. The set of contour pixels are named *P_1_, P_2…_P_N_*.
2. The middle point of the intersection line formed by the wrist and the bottom line of the palm image is defined as *W_m_*.
3. By using Equation (3), the Euclidean distance between each contour pixel and the wrist middle point *W_m_* is calculated. These distances are adopted to construct a distance distribution profile whose shape is similar to the geometric shape of a palm. Figure 4b shows the distance distribution profile. The distance profile has five local maximums corresponding to fingertip locations, and four local minimums corresponding to finger-web locations. After experimenting with many palm images, this study finds that the four minimum locations are the same as the finger-web locations regardless of the palm position and rotation angle in each image. With this characteristic, the four finger webs, *FW*_1_, *FW*_2_, *FW*_3_, and *FW*_4_, can be accurately located by referencing the four local minimums in the distance distribution profile.
4. Contour- and curvature-based outstretched hand detection is employed to locate finger webs. The locations of the four finger webs, *FW*_1_, *FW*_2_, *FW*_3_, and *FW*_4_, are shown in Figure 5a.
5. Since the rotation angle of the palm region is different in each image, the rotation angle must be removed. Line $\bar{FS}$ is formed using finger webs *FW*_2_ and *FW*_4_ as shown in Figure 5a. The angle θ between $\bar{FS}$ and a horizontal line is calculated using Equation (4). The resulting image rotated with angle θ is shown in Figure 5b. Figure 5c shows $\bar{FS}$ rotated to be horizontal.
6. A square ROI is defined by selecting the second- and fourth-finger webs, *FW*_2_ and *FW*_4_. *FWm* is the middle point of *FW*_2_ and *FW*_4_. The square region is determined by the corners *R_1_*, *R_2_*, *R_3_*, and *R_4_*. The top side $\bar{R_{1}R_{2}}$ is parallel to $\bar{FS}$ and the distance between them is a quarter length of the line $\bar{{FW}_{2}{FW}_{4}}$. $\bar{R_{1}R_{2}}$ is calculated as (3/2) × $\bar{{FW}_{2}{FW}_{4}}$. The corner *R_1_* is then redefined as the original coordinate (0, 0) of the ROI. The located ROI is shown in Figure 5c. The ROI image, which is the square region enclosed by corners *R_1_*, *R_2_*, *R_3_*, and *R_4_*, is cropped from the palm or palm-dorsum image.
  (3) $$D_{E}\text{(}i)=\sqrt{{\left(X_{W_{M}}{-X}_{P_{i}}\right)}^{2}+{\left(Y_{W_{M}}{-Y}_{P_{i}}\right)}^{2}}$$

where (*X_WM_*, *Y_WM_*) are the coordinates of the wrist middle point *W_M_* , (*X_Pi_*, *Y_Pi_*) are the coordinates of the *i*-th pixel of the contour set, and *D_E_(i)* is the Euclidean distance between the wrist middle point *W_M_* and the *i*-th pixel of the contour set.

θ = *tan*^−1^[(*Y_FW2_* − *Y_FW4_*)∕(*X_FW2_* − *X_FW4_*)] (4)

where *θ* is the angle between line $\bar{FS}$ and a horizontal line, (*X_FW2_*, *Y_FW2_*) are the coordinates of *FW*_2_, and (*X_FW2_*, *Y_FW4_*) are the coordinates of *FW*_4_.

**Figure 6:**
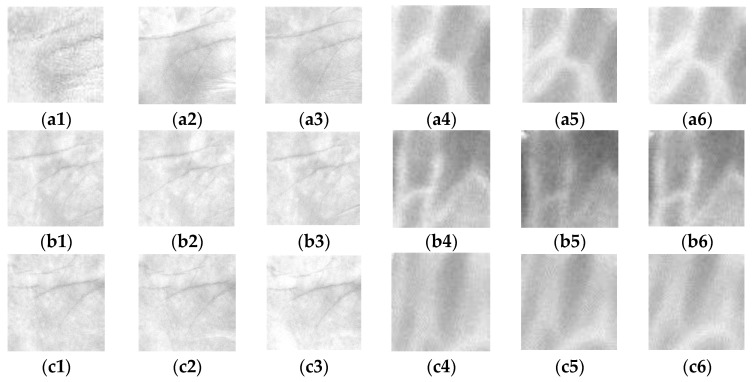
(**a1**)–(**a3**), (**b1**)–(**b3**) and (**c1**)–(**c3**) show the ROIs of palm regions cropped from Figure 2a1–a3,b1–b3,c1–c3, respectively; (**a4**)–(**a6**), (**b4**)–(**b6**) and (**c4**)–(**c6**) show the ROIs of IR palm-dorsum regions cropped from Figure 2a4–a6,b4–b6,c4–c6, respectively.

The ROIs for palm regions are located and cropped. The ROIs for palm regions are normalized to 256 × 256 pixels. The ROIs for palm-dorsum regions are normalized to 64 × 64 pixels. Figure 6 shows the different ROIs for palm and palm-dorsum regions cropped from Figure 2. The ROIs are located at almost exactly the same region. Therefore, the problem of varied palm locations and rotation angles can be solved.

## 3. Image Fusion Based on DWT and IDWT

Image fusion has been employed in diverse fields such as computer vision, remote sensing, medical imaging, and satellite imaging. Irrespective of the field, the aim of image fusion is the same that is to create more useful information from two single images.

DWT is a useful technique for numerical and functional analysis. It has long been used as a method of image fusion [25], and its practical applications can be found in digital communication, data compression, and image fusion. A key advantage of DWT over Fourier transforms is that it captures both frequency and location information. The proposed method fuses images by using DWT and IDWT with a novel hybrid fusion rule at different decomposition levels in wavelet-based.

For the described DWT, some necessary signals and filters must first be defined. Signal *x_i_* is the input signal. Signal *y_o_*, which includes *y_low_* and *y_high_*, is the output signal. Filter *l*, as expressed in Equation (5), is a low pass filter that filters out the high frequency of the input signal and outputs the low frequency signal called approximation coefficients. Filter *h*, as expressed in Equation (6), is a high pass filter that outputs the high frequency signal called detail coefficients. Variables *k* and *n* are the *k-th* and *n-th* data of the signal, respectively. The filter outputs are downsampled by two with the downsampling operator ↓:

(5) $$y_{low}[n]=\sum_{k=-\infty }^{\infty }x_{i}[k]l[2n-k]$$

(6) $$y_{high}[n]=\sum_{k=-\infty }^{\infty }x_{i}[k]h[2n-k]$$

**Figure 7:**
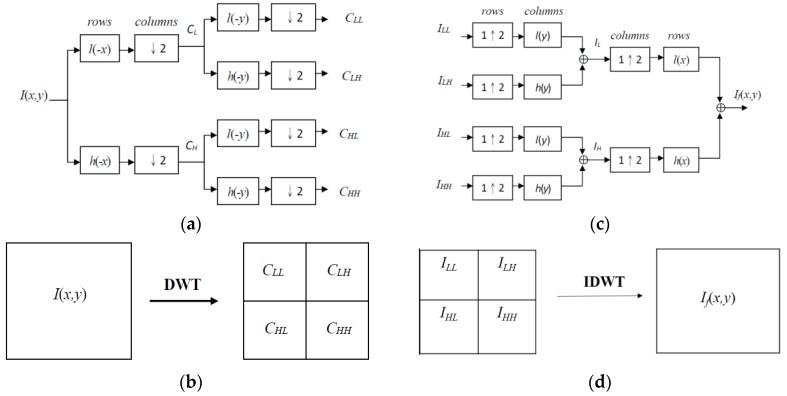
(**a**) One-stage structure of the two-dimensional DWT; (**b**) Relative locations of decomposition coefficient matrices in the two-dimensional DWT; (**c**) One-stage structure of the two-dimensional IDWT; (**d**) Relative locations of fusing coefficient matrices in the two-dimensional IDWT.

The DWT of signal *x_i_* is calculated by passing *x_i_* through a series of filters. The signal is decomposed using a low pass filter and a high pass filter simultaneously. The decomposition is repeated to further increase the frequency resolution, and the approximation coefficients are decomposed with the high and low pass filters and the downsampling. Figure 7a shows the one-stage structure of the two-dimensional DWT where *l*(−*x*) and *l*(−*y*) are the low pass decomposition filters, *h*(−*x*) and *h*(−*y*) are the high pass decomposition filters, and *C_LL_*, *C_LH_*, *C_HL_*, and *C_HH_* are the decomposition coefficient matrices. Figure 7b shows the relative locations of decomposition coefficient matrices in the two-dimensional DWT.

Once the coefficients are merged, the final fused image is achieved using IDWT. Figure 7c shows the one-stage structure of the two-dimensional IDWT. The fused image is denoted by *I_f_* (*x, y*). Figure 7d shows the relative locations of the fusing coefficient matrices in the two-dimensional IDWT. *I_LL_*, *I_LH_*, *I_HL_*, and *I_HH_* are the fused coefficient matrices.

### Wavelet-Based Image Fusion of Palmprints and Vein Patterns

Palmprints have low gray levels in palm images, whereas vein patterns have high gray levels in palm-dorsum images. To make the gray-level properties of palmprints consistent with those of vein patterns, the gray levels of the palm images are reversed. Thus, inverted palm images have high gray-level palmprints with low gray-level backgrounds.

**Figure 8:**
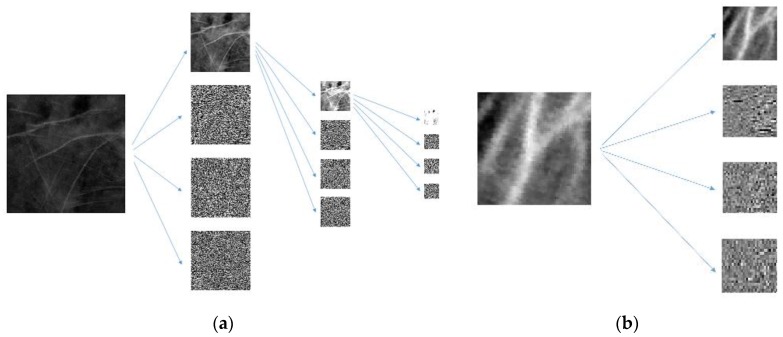
(**a**) Three levels of DWT in palmprint ROI decomposition; (**b**) One level of DWT in vein pattern ROI decomposition.

In addition, the ROI sizes for palmprints and vein patterns are different. To address this problem, palmprint ROIs are decomposed using two-dimensional DWTs three times to obtain first-, second-, and third-level coefficients with the sizes of 128 × 128, 64 × 64, and 32 × 32 pixels, respectively (see Figure 8a). Vein pattern ROIs are decomposed using a two-dimensional DWT to obtain the first-level coefficient with a size of 32 × 32 pixels (see Figure 8b). The size of the third-level coefficient for the palmprint ROI is the same as the size of the first-level coefficient for the vein pattern ROI. The two-dimensional DWT used in the proposed method is the Haar filter, which includes lowpass filter *l* and highpass filter *h*.

**Figure 9:**
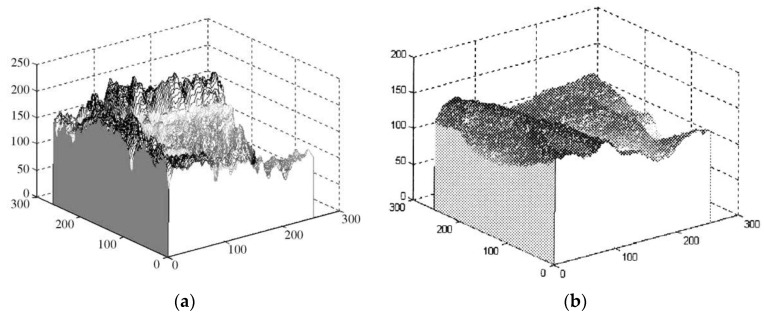
3D profile of palmprint and vein pattern ROIs. The vertical axis represents the gray level of pixels. The other two axes represent the coordinates of pixels. (**a**) The profile of the palmprint ROI shows a sudden change in the gray levels of adjacent pixels near the principal palmprint, which possesses high frequency; (**b**) The vein pattern profile demonstrates that the gray levels of the vein pattern vary smoothly. The vein pattern has a low frequency.

By analyzing the three-dimensional (3D) profiles of the palmprint and vein pattern ROIs, it is revealed that palmprints and vein patterns possess different characteristics. The 3D profile of the palmprint ROI shows a sudden change in the gray levels of adjacent pixels near the principal palmprint, which possesses a high frequency as shown in Figure 9a [12]. In contrast, the 3D profile of the vein pattern ROI demonstrates that the gray levels of the vein pattern varies smoothly. The vein pattern has a low frequency as shown in Figure 9b [17].

Coefficient fusion is the key step in image fusion based on wavelets. Many coefficient fusion rules have been presented including maximum, minimum, average, weighted, down-up, and up-down [25]. According to an analysis of the 3D profiles for palmprint and vein pattern ROIs, the proposed method introduces a hybrid fusion rule consisting of average and maximum fusion rules. The hybrid fusion rule applies the average rule to combine the approximation coefficients and the maximum rule to combine the detail coefficients. The hybrid fusion rule is named the *Avg-Max* fusion rule and is expressed as follows:

(7) $$I_{f}\;\left(x,\;y\right)=\{\begin{array}{l} I_{p}\left(x,\;y\right)+I_{v}(x,\;y))/2\;\left(x,\;y\right)\in \text{approximation subband}\\ max\;(I_{p}\left(x,\;y\right),\;I_{v}(x,\;y))\left(x,\;y\right)\in \text{detail subband} \end{array}$$

where *I_f_* (*x, y*) is the coefficient value of the fused image at pixel (x, y), *I_p_*(*x, y*) is the coefficient value of the palm image, and *I_v_*(*x, y*) is the coefficient value of the palm-dorsum vein image.

**Figure 10:**
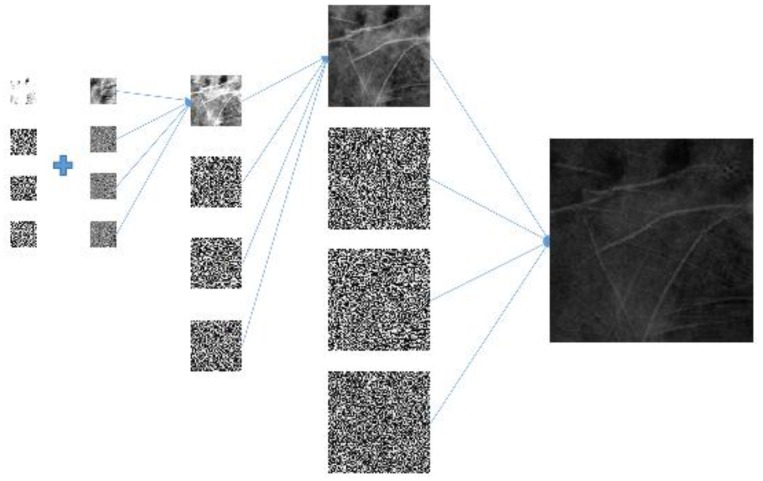
Three stages of IDWT fused image composition.

Since the sizes of the palmprint and vein pattern ROIs are different, the proposed method adopts the different resolution fusion scheme [26] to fuse palmprint and vein pattern ROIs at the third and first levels, respectively. The size of the third-level coefficient of the palmprint ROI is the same as the size of the first-level coefficient of the vein pattern ROI which is 32 × 32 pixels. The proposed method applies the novel hybrid fusion rule to combine the 32 × 32 coefficients and performs IDWT to fuse the 64 × 64 image. IDWT is then performed again with the remaining palmprint coefficients. The final fused 256 × 256 image is shown in Figure 10. We can observe that the fused image retains the high frequency palmprint and low frequency vein pattern information.

## 4. Feature Extraction

### 4.1. Enhancement of Line Features

**Figure 11:**
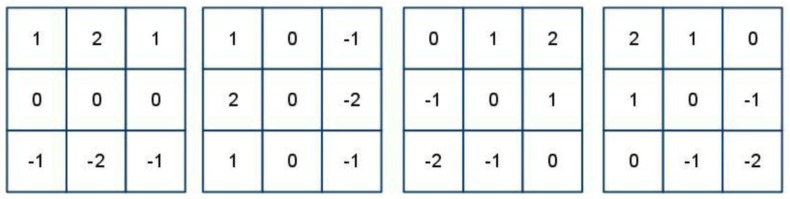
Four 3 × 3 Sobel operators in different orientations.

Feature extraction is essential for recognition systems. To extract features, the Sobel operator is employed to enhance the LLFs of the fused ROIs. The Sobel is a well-known filter used to detect the discrete directional gradient that includes 0, 45, 90, and 135 degrees. The four directional operators are shown in Figure 11.

**Figure 12:**
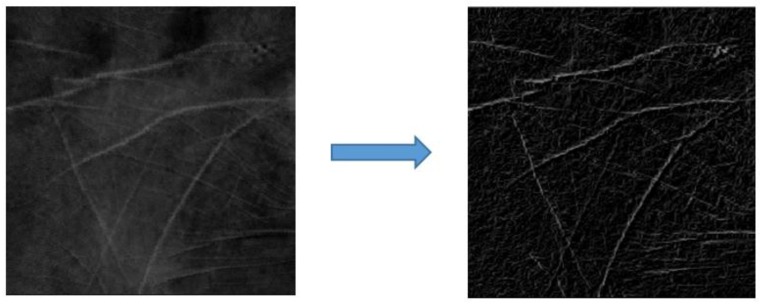
Example illustrating the result from applying a Sobel operator.

The fused ROIs are filtered using Sobel operators in the different orientations used to emphasize the edges in specified directions. For example, the Sobel operator of 0 degrees is used to emphasize horizontal line edges. The four Sobel operators are applied to convolute each pixel in the fused ROIs and generate four values. The maximum value from these four generated values is selected as the gray level of pixels in the enhanced ROI. The enhanced ROI is shown in Figure 12.

### 4.2. LLF Extraction

Because the line edges of the fused ROIs are enhanced using Sobel operators, it is crucial to accurately extract the LLFs. The capture of palm and palm-dorsum images at different times induces a variable environmental condition of different images. Therefore, a fixed threshold is not suitable to extract LLFs. The proposed method adopts the iterative threshold selection technique [23] to extract the line feature pixels of enhanced ROIs. The procedures are outlined as four steps as will be briefly described as follows:

Step 1. Randomly initialize the threshold *Th*(1) (between the range of 0 to 255) to segment the image into the object and the background.
Step 2. Apply Equations (8) and (9) to iteratively compute μ*_B_*(*i*) and μ*_o_*(*i*) as the means of the background and object gray levels at the *i-th* iteration, respectively. The threshold *Th*(*i*) used to segment images into the background and object is determined in Step 3 of the previous iteration by using Equation (10).
  (8) $$\mu_{B}=\sum_{(i,j)\in background}f(i,j)/N_{B}$$
  (9) $$\mu_{o}=\sum_{(i,j)\in object}f(i,j)/N_{o}$$

where *N_O_* and *N_B_* denote the pixel numbers of the object and the background, respectively.

Step 3.  
Set *Th*(*i* + *1*) = (μ*_B_*(*i*) + μ*_o_*(*i*))/2 (10)

where *Th*(*i* + *1*) is the new threshold for the next iteration.

Step 4. If *Th*(*i* + *1*) = *Th*(*i*), then terminate; otherwise return to Step 2.

The threshold *Th* is thereby determined. The enhanced ROIs are thereby transformed into binary values. Binary images including only LLFs are called line-like images (LLIs). Detailed procedures can be found in [23].

### 4.3. Multiresolution Analysis with a Multiresolution Filter

The multiresolution analysis of signals has been proven effective in extracting local features. In addition, a multiresolution representation provides an uncomplicated hierarchical structure for interpreting image information. Information at different resolutions of an image generally represents different physical structures. A coarse resolution image is viewed as though seeing an object from far away. It possesses gross information and is less sensitive to noise and disturbances. By contrast, a fine resolution image provides the capacity for seeing an object at closer distances revealing detailed information that is more sensitive to noise and disturbances.

**Figure 13:**
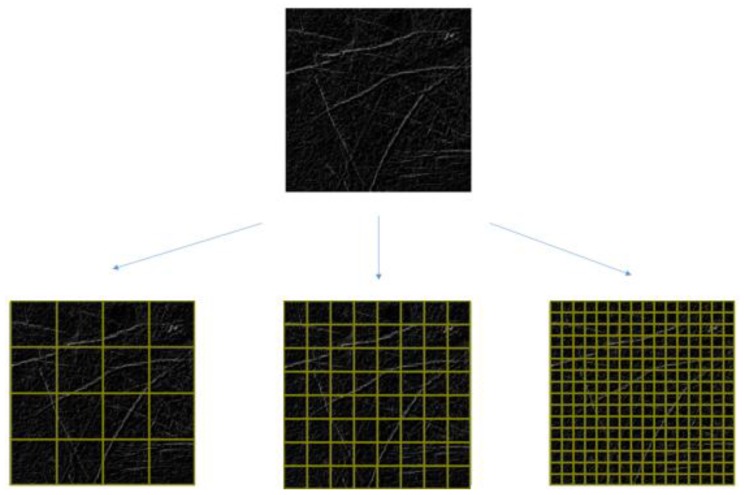
Example of nonoverlapping blocks.

The proposed method employs a feature-pass filter, presented as FPF in Equation (11), as a multiresolution filter to decompose LLIs into multiresolution. Figure 13 shows that with an LLI of *m* × *m* pixels, the LLI is decomposed into *n* × *n* nonoverlapping blocks and the resolution of each block contains (*m/n*) × (*m/n*) pixels. The feature-pass filter calculates the number of LLF pixels in each block as a feature vector. The multiresolution filter proves clearly that it can solve the problem of offset in the position of the palm across different images.

(11) $${FPF}^{\left(n\right)}(p,q)=\sum_{x=1}^{m/n}\sum_{y=1}^{m/n}LLI(x,y)$$

where *FPF* ^(*n*)^(*p,q*) is the number of LLF pixels inside each nonoverlapping block of (*m/n*) × (*m/n*) pixels, *x and y* represent the horizontal and vertical coordinates respectively, and *x, y*∈{ 1,2, ..., (*m*/*n*)}. LLI(*x, y*) is a binary image with LLFs. At the *nth* level of resolution, *p* and *q* represent the horizontal and vertical coordinates and *p, q*∈{ 1,2, ..., *n*}. These numbers are recorded as feature vectors for later verification.

## 5. Verification

### Support Vector Machine

The SVM is a well-known supervised learning model and a useful scheme for data classification. SVM essentially searches for the optimal separating hyperplane that is a linear or nonlinear decision boundary separating two classes and leaves the largest margin between the vectors of the two classes. An optimal separation is achieved by the hyperplane that has the largest distance to the nearest training data point of any class. In general, a larger margin causes a lower generalization error in the classifier.

The simplest model of an SVM classifier is the *maximal margin classifier*. It attempts to insert a linear plane between two classes and to orient this plane such that the margin 2/||*w*|| is maximized. The middle of the margin is named the optimal hyperplane. The data points lie on the boundaries and those closest to the margin are called support vectors. The equation is expressed as:

(12) $$\begin{array}{c} y_{i}\;\left(w^{T}x_{i}+b\right)\;\geq \;1\text{ for i}=1,\;2,\;3,\;\ldots \ldots ,N\text{ and }x_{i}\in A\cup B\\ min_{\;w,b}\;(\frac{1}{2}||w|{|}^{2}) \end{array}$$

where *x_i_* is the support vector belonging to class *A* and *B* with *y_i_*∈{−1, 1}, *b* denotes the location of the hyperplane relative to the origin, *N* is the number of support vectors, and 2/||*w*|| is the margin between the hyperplane and support vectors.

However, this method is only suitable for two separable classes. If two classes cannot be completely separated, this method is not feasible. Because real-life classification problems are difficult to solve using a linear classifier, an improved version with a nonlinear decision surface is necessary. This can be achieved using the kernel scheme, which applies a nonlinear kernel function to replace the linear kernel function in Equation (12). Accordingly, the applied kernel function such as linear, polynomial, Gaussian, or radial basis functions, plays a crucial role in the classification of SVMs. This is expressed as:

(13) $$\begin{array}{c} y_{i}\;\left(w^{T}\Phi \left(x_{i}\right)+b\right)\;\geq \;1-\xi_{i}\text{ for }i=1,\;2,\;3,\;\ldots \ldots ,N\text{ and }x_{i}\in A\cup B,\;\xi_{i}>\;0\\ \min_{w,b,\xi }(\frac{1}{2}w^{T}w)+C\sum_{i=1}^{N}\xi_{i}\;i=1,\;2,\;3,\;\ldots \ldots ,N \end{array}$$

where *Φ* denotes the function-mapping support vectors *x_i_* into a higher dimensional space, *ξ* is the slack variable controlling the misclassification caused by linearly nonseparable classes, and *C* represents a parameter controlling the tradeoff between classification errors and the margin in the training set. Detailed information on SVMs is described in [27].

In this study, the feature vectors of the LLI multiresolution representation are used as the input test sample for the SVM to verify as genuine or an impostor. In our work, we adopt the well-known Library for Support Vector Machines (LIBSVM) tool developed by C. Chang *et al.* in [28] as the verification method. The radial basis function, expressed in Equation (14), is used as the kernel function in the SVM classification process:

(14) $$K\left(x_{i},\;x_{j}\right)\;=exp(―||\;x_{i}―x_{j}\;|{|}^{2}∕\;2\sigma^{2})$$

where σ is a kernel parameter.

In addition, k-fold cross validation is employed to construct the parameter library with (*k −* 1)/*k* positive training samples of all palm images and with (*k* − o)/*k* negative training samples randomly selected from the images of other palms. To verify the validity of the proposed method, the remaining 1/*k* images of a palm are used as positive testing samples and the 1/*k* images randomly selected from the images of other palms are used as negative testing samples.

## 6. Results

The experimental platform is a personal computer with a 64-bit Microsoft Windows 7 operating system, an Intel(R) Core(TM) i7 CPU 2.80 GHz processor, and 4 GB RAM. The developing tools used are Microsoft Visual Studio C++ (2010) with OpenCV library and MATLAB 2013a.

### 6.1. Data Collection

**Figure 14:**
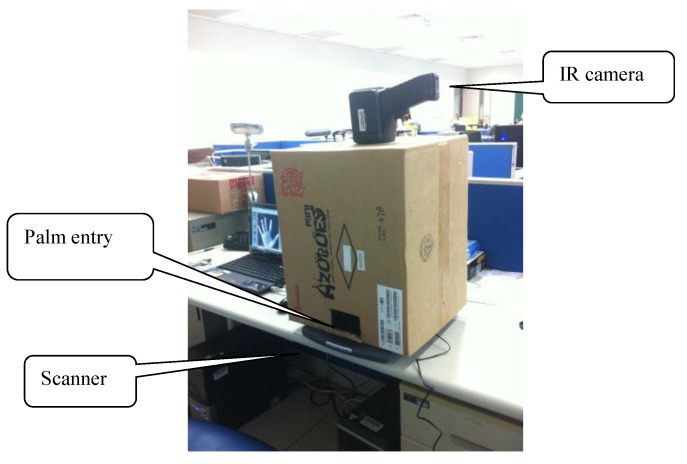
The configuration of the image collection system.

Experimental data was collected using the digital scanner and the infrared camera. The total number of palm images is 3000, and the total number of palm-dorsum images is 3000. The palm and palm-dorsum images were collected from 100 people with each having 30 images captured using the scanner and infrared camera, respectively. The original size of palm images is 845 × 829 pixels with 256 gray levels, and the original size of palm-dorsum images is 320 × 240 pixels with 256 gray levels. Figure 14 shows the configuration of the image collection system.

### 6.2. Experimental Results

The digital scanner used the capture palmprint images is an Avision FB1200 (Avision Inc., Hsinchu, Taiwan). The IR camera used to capture IR palm-dorsum images is Thermo GEAR G100 that was produced by the NEC Corporation (Tokyo, Japan). The following descriptions are their specifications:

Digital scanner:
Document feeding modeFlatbedLight SourceLEDOptical Resolution (dpi)Up to 2400 dpiGrayscale mode8bits outputColor mode24bits outputInterfaceUSB 2.0Output Format: JPEG image format

IR camera:
Infrared Detector: Uncooled Focal Plane Array (microbolometer)Noise equivalent temperature difference (NETD) : 0.08 °C (at 30 °C, 60 frames/s.)Accuracy: ±2 °C or ±2% of Reading, whichever is greaterMeasuring Range: −40 °C to 500 °CSpectral Range: 8 to 14 μmThermal Image resolution: 320(H) × 240(V) pixels Field of View: 32°(H) × 24°(V) (standard lens F.L. = 14 mm)Spatial Resolution (IFOV): 1.78 mradA/D Resolution: 8 bitsOperating Temperature/Humidity: −15 °C to 50 °C, <90% RHOutput Format: JPEG image format

In the experimental results, the false rejection rate (FRR) and the false acceptance rate (FAR) are used as two benchmarks to evaluate the performance of the proposed method. The lower the values of the FRR and FAR are, the higher the performance is of the proposed method. The FRR and FAR are defined as follows:

FRR = (NFR/NPT) × 100%
(15)

FAR = (NFA/NNT) × 100%
(16)

where NPT is the number of positive test samples, NNT is the number of negative test samples, NFR is the number of false rejections, and NFA is the number of false acceptances.

**Figure 15:**
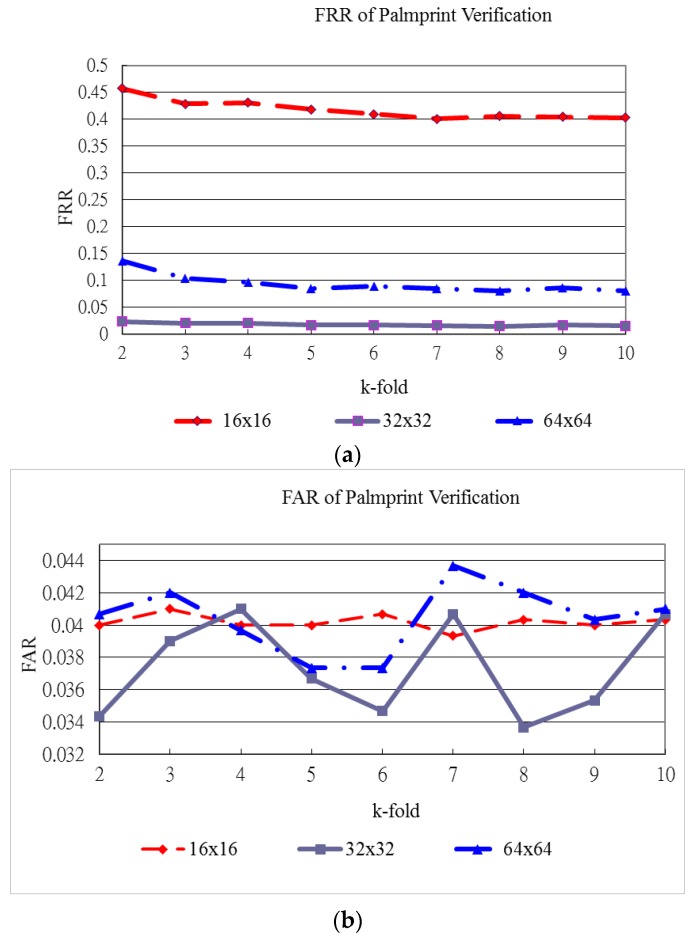
FRR and FAR for palmprint verification. (**a**) FRR of palmprint verification; (**b**) FAR of palmprint verification.

**Figure 16:**
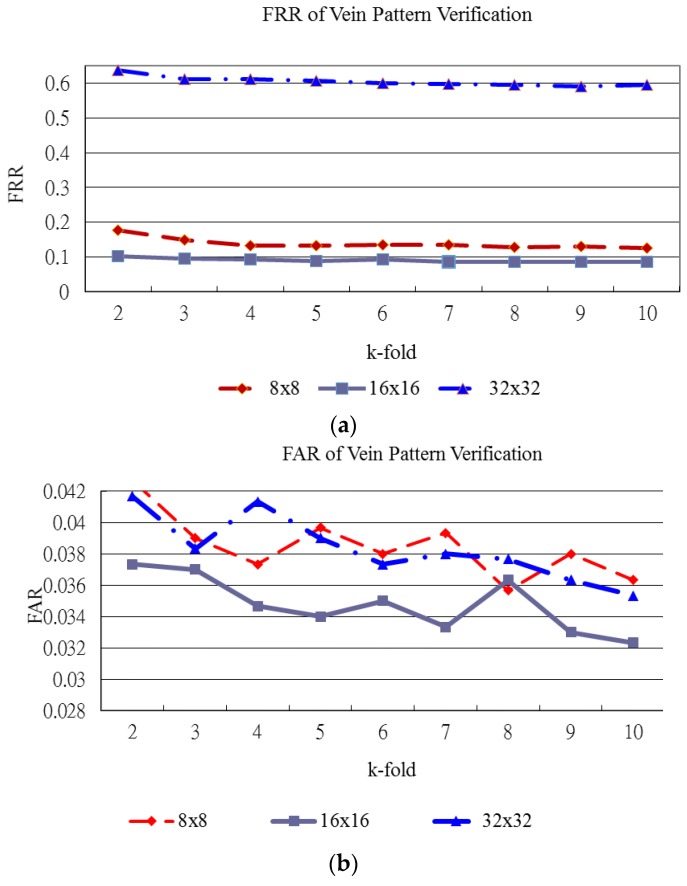
FRR and FAR for vein pattern verification. (**a**) FRR of vein pattern verification; (**b**) FAR of vein pattern verification.

**Figure 17:**
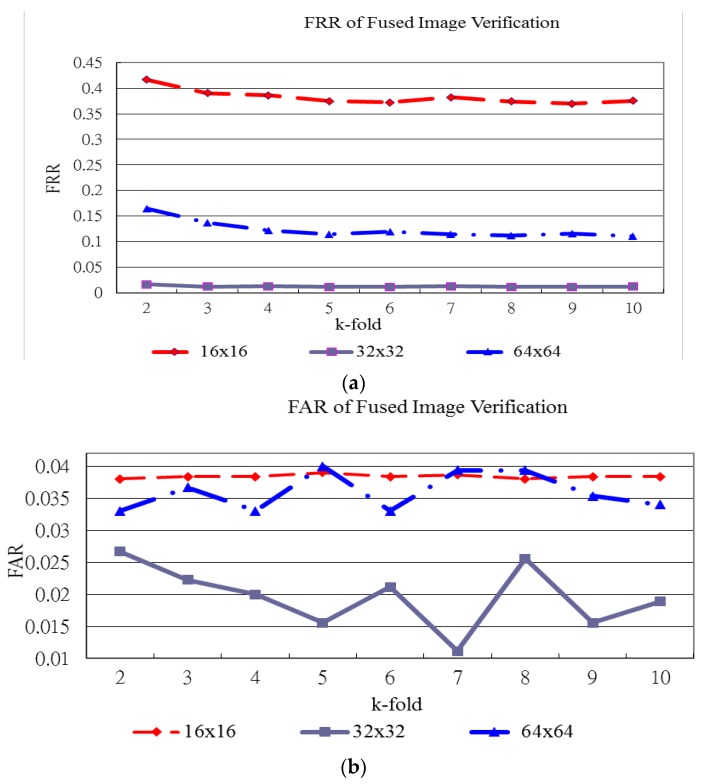
FRR and FAR for fused image verification. (**a**) FRR of fused image verification; (**b**) FAR of fused image verification.

Figure 15, Figure 16 and Figure 17 show the verification results of plamprint, vein pattern and fused (palmprint + palm-dorsum) images, respectively. These three figures exhibit the different performance between single modal and bimodal biometric. The vertical axes represent the FRR and FAR in Figure 15a,b, respectively. The horizontal axe represents the k-holds in both Figure 5a,b. The vertical and horizontal axes of Figure 16 and Figure 17 are defined as same as of Figure 15.

Figure 15 shows the FRR and FAR for palmprint verification generated by various block sizes and k-holds. Figure 15a shows that the FRR generated by the 32 × 32 block size produces the lowest curve for all k-holds. The lowest FRR for palmprint verification reaches 1.43% for the 32 × 32 block size with 8-fold cross validation as shown in Figure 15. In this case, the FAR for palmprint verification is the lowest at 3.37%.

Figure 16 shows the FRR and FAR for vein pattern verification generated by various block sizes and k-holds. Figure 16a shows that the FRR generated by the 16 × 16 block size produces the lowest curve for all k-holds. The lowest FRR and FAR for vein pattern verification are 8.53% and 3.30%, respectively. The highest performance for vein pattern verification is achieved with the 16 × 16 block size and 9-fold cross validation as shown in Figure 16.

**Figure 18:**
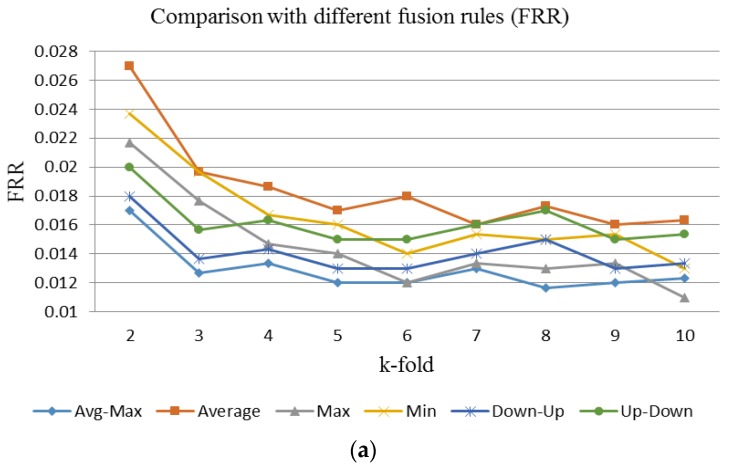

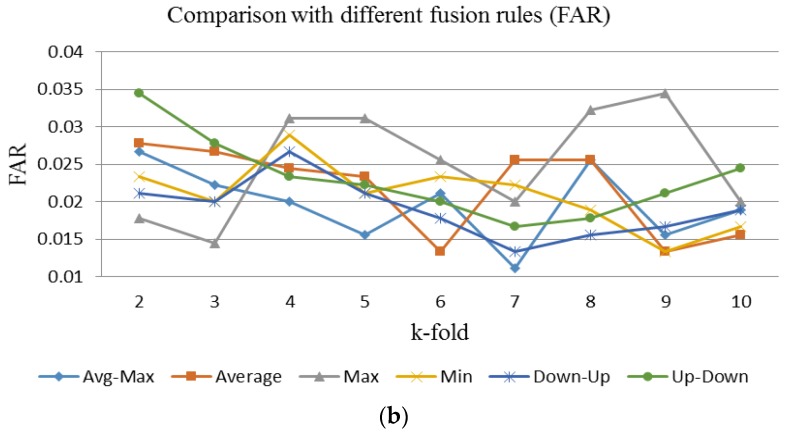
The FRR and FAR of fused images fused by using the proposed hybrid fusion rule, Avg-Max, and the other fusion rules with 32 × 32 block size and various k-holds. (**a**) FRR of fused image verification with different fusion rules; (**b**) FAR of fused image verification with different fusion rules.

Figure 17 shows the FRR and FAR for fused image verification generated using various block sizes and k-holds. Figure 17a shows that the FRR generated using the 32 × 32 block size produces the lowest curve for all k-holds. In high-security demand applications, the lowest FRR is needed. The lowest FRR for fused image verification is 1.20% with a FAR of 1.56%. This performance is achieved with the 32 × 32 block size and 9-fold cross validation. For convenient and suitable security demand applications, the lower FAR is needed. The lowest FAR for fused image verification is 1.11% with an FRR of 1.30%. This performance for fused image verification is achieved with the 32 × 32 block size and 7-fold cross validation. Figure 18 shows the FRR and FAR of fused images fused by using the proposed hybrid fusion rule, Avg-Max, and the other fusion rules with 32 × 32 block size and various k-holds. Figure 18a shows the FRR generated by using the Avg-Max fusion rule is the lowest between the range from 2-hold to 9-hold. Figure 18b illustrates the FAR generated by using the Avg-Max fusion rule is lower than that by using some fusion rules. However, it is not the lowest. The main reason is the negative training and testing samples are randomly selected from the image database.

The performance of bimodal biometric verifications is not easily compared since there is no public standard database. However, we summarize some results of verification methods and the proposed method which fuses palmprint and vein pattern images in Table 1, which shows the performance of the proposed method is comparable to other verification methods.

**Table 1:** Comparison of palmprint or palm vein verification methods and the proposed method which fuses palmprint and vein pattern images.

Reference Paper	Database		Best Results		
	Number of Palm Images	Number of Palms	Accuracy Classification Rate (%)	FRR (%)	FAR (%)
[2] (Dataset I)	1300	100		0.49	0.50
[2] (Dataset II)	5018	386		0.57	0.57
[4]	100		95.00		
[5]	3056	191		1.00	0.03
[10]	9000	300	99.04		
[12]	4800	160	99.25	0.75	0.69
[13]	1500	50	98.00	2.00	2.00
[17]	960	30		2.30	2.30
[19]*	1440	120		0.32	0.10
[29]	200	20	97.00		
[30]	200	100	91.00		
[31]	7200	200		1.64	1.64
[32]		100	99	1.00	0.29
Proposed method	6000	100	98.8	1.20	1.56

* FAR and FRR are estimated from Figure 11 in [19] by eye.

Table 1 indicates that its performance is slightly higher than that of the verification methods proposed by [4,13,17,29,30]. By contrast, the performance of the verification methods proposed by [2,5,10,12,19,31,32] is slightly higher than that of the proposed method. Some possible explanations for this are as follows. Lin *et al.* [10] did not discuss FARs in their study. In real-world applications both FRR and FAR must be low, as a low FRR implies that as few legitimate users as possible are rejected by a system, while a low FAR implies that as few impostors as possible are falsely accepted by a system. In [12], Lin *et al.* extracted multiple features for verification. In practice, more features produce a higher accuracy rate. The palm images used in Huang *et al.* [2] and Lu *et al.* [5] were captured using pegs or other devices to constrain the palm position. As such, the movement of ROIs in palm images was reduced and hence leading to a higher accuracy rate. Raghavendra *et al.* [31] applied multispectral six-band palmprint images for verification. A larger number of different band images carry more information that can be used for verification and hence leading to a higher accuracy rate. Vaidya *et al.* [32] used visible light and near infrared (NIR) cameras to capture the palmprint and palm vein images, respectively. The sensor material of these two cameras is complementary metal-oxide semiconductor (COMS). The images size of palmprint and palm vein images are 1024 × 768 pixels. They are more than that of used images to evaluate the proposed method. In practice, a larger size image has more information and thus it can achieve a higher accuracy rate of verification. The reason why Wang *et al.* [19] report a higher accuracy rate of verification is similar to that of Vaidya *et al.* [32]. In additional, the palmprint and palm vein images used in Wang *et al.* [19] were captured using docking devices to constrain the palm position. This substantially improves the recognition performance significantly. The palm vein images used in [19] and [32] were captured from palm side by an NIR camera. 

**Table 2:** The detailed differences between the two capturing devices and scenarios used in reference [19] and the proposed method.

	Proposed Method		Reference [19]	
Used image	Palmprint image	Palm-dorsum vein image	Palmprint image	Palm vein image
Image captured from	Palm	Palm-drosum	Palm	Palm
Capturing device	Scanner	Infrared camera	camera	Near infrared camera
Capturing scenarios	Peg-free	Peg-free	Use docking device	Use docking device
Sensor material	CMOS	Microbolometer	CMOS	CMOS
Sensor type	Linear	Focal Plane Array	Focal Plane Array	Focal Plane Array
Sensor spectral response	380–750 nm	8–14 μm	380–750 nm	750–1000 nm
Imaging mechanism	Detect reflected visible spectrum	Detect radiated IR spectrum	Detect reflected visible spectrum	Detect reflected NIR spectrum
Image size	845 × 829	320 × 240	768 × 576	768 × 576

On the contrary, the pal[mprint and palm-dorsum vein image were collected by visible light scanner and infrared (IR) camera. Each image was captured in peg-free scenarios to improve the user-friendliness. Visible light scanner and infrared (IR) cameras are two different kinds of capturing devices. The sensor material of scanner is COMS and that of IR camera is microbolometer. Thus the physiological characteristics and image size of palmprint and palm-dorsum vein images are quite different. The differences between the two capturing devices used in the proposed method are much more than those used in reference [19]. The detailed differences between the two capturing devices and scenarios used in reference [19] and the proposed method are summarized in Table 2.

## 7. Conclusions

In this paper, fused images of palmprints and palm-dorsum vein patterns are used to verify the identity of individuals. The experimental results show that the proposed biometric verification system is robust and reliable. The findings of this research can help extend bimodal biometric verification technology to security access control systems and bio-cryptosystems [33,34].

There are five advantages in our proposed method. First, no docking devices or fixed pegs are needed while acquiring palm images, which makes the personal verification device easier and more convenient for users. Second, the low-resolution images are used to verify and result in a smaller database. Third, the threshold values to binarize the original image to the background and palm region are automatically set. Hence, the palm region is segmented adaptively using the proposed thresholding technique. Fourth, according to the palmprint and vein pattern characteristics, this paper proposes a novel hybrid fusion rule, Avg-Max, to fuse the different coefficients decomposed by DWT. In addition, the palmprint and vein pattern images are of different sizes, yet the proposed method combines the different coefficients at different decomposition levels with coefficients of the same size. Finally, the fused image creates richer and more useful information than each individual image and the dimensions of the feature vectors are the same as in each individual image. 

As with most biometric verification methods, the proposed method has some operational limitations. Because the IR camera used in this study has a low resolution and sensibility, this limits the accuracy of biometric verification. A high performance IR camera should be used to capture high-quality and more discriminative images. Furthermore, there may exist other effective feature extraction methods that could obtain more information from palm and palm-dorsum images. In addition, a biometric verification method combining additional biometric features such as palm geometry, fingerprints, or palm creases could increase verification accuracy. Finally, most biometric features vary with the age of the person, an improved biometric verification method would be capable of predicting feature variations to maintain accuracy.
